# Supplementary material for: Tailoring π-conjugation and vibrational modes to steer on-surface synthesis of pentalene-bridged ladder polymers
Source: Nat Commun. 2020 Sep 11;11:4567. doi: 10.1038/s41467-020-18371-2 (PMC7486926; doi:10.1038/s41467-020-18371-2)
Supplement: Supplementary file 1 — Supplementary Information [file 41467_2020_18371_MOESM1_ESM.pdf]

Supplementary Information for:

Tailoring π-conjugation and vibrational modes to steer on-surface synthesis of pentalene-bridged ladder polymers

Bruno de la Torre[1,2♦], Adam Matěj[1,2♦], Ana Sánchez-Grande[3♦], Borja Cirera[3], Benjamin Mallada[1,2], Eider Rodríguez-Sánchez[3], José Santos[3,4], Jesús I. Mendieta-Moreno[2], Shayan Edalatmanesh[1,2], Koen Lauwaet[3], Michal Otyepka[1], Miroslav Medveď[1], Álvaro Buendía[5], Rodolfo Miranda[3,5], Nazario Martín[3,4]*, Pavel Jelínek[1,2]* and David Écija[3]*

[1]Regional Centre of Advanced Technologies and Materials, Palacký University, Šlechtitelů 27, 78371 Olomouc, Czech Republic.
[2]Institute of Physics, The Czech Academy of Sciences. Cukrovarnická 10, 162 00 Prague 6, Czech Republic.
[3]IMDEA Nanociencia, C/ Faraday 9, Ciudad Universitaria de Cantoblanco, 28049, Madrid, Spain.
[4]Departamento de Química Orgánica, Facultad de Ciencias Químicas, Universidad Complutense, 28040 Madrid, Spain.
[5]Departamento de Física de la Materia Condensada, Universidad Autónoma de Madrid, Cantoblanco, Madrid, Spain.

*Correspondence to: jelinekp@fzu.cz (P.J.), nazmar@ucm.es (N.M.), david.ecija@imdea.org (D.E.)

This PDF file includes:

Supplementary Fig. 1 | Polymerization evolution of 4BrAn precursor described in the main text.

Supplementary Fig. 2 | Scheme of QM/MM region and reaction coordinates.

Supplementary Fig. 3 | Scheme of calculated reaction pathways of bisanthene polymer.

Supplementary Fig. 4 | Scheme of calculated reaction pathways of anthracene polymer.

Supplementary Fig. 5 | List of available bending vibrational modes in anthracene and bisanthene polymers and their comparison with the reaction coordinates.

Supplementary Fig. 6 | Experimental and theoretical bond-length analyses.

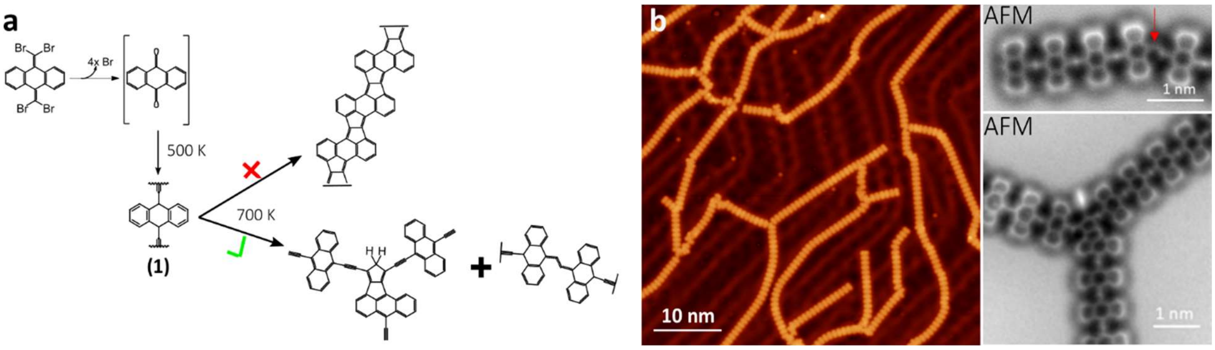

Supplementary Fig. 1 | Polymerization evolution of 4BrAn precursor described in the main text. a, Scheme of the reaction sequence of **4BrAn** precursor after being deposited on Au(111), annealed to 500 K to afford polymer **1.** Further annealing up to 700 K gives rise to inter-polymeric fusion, carbon atom addition and intra-polymeric warping instead of pentalene formation. **b,** STM overview (left panel) of **4BrAn** precursor after annealed to 700 K on Au(111), and AFM high-resolution detail of an intra-polymeric warping (top right panel) and inter-polymeric (right bottom panel) fused structures.

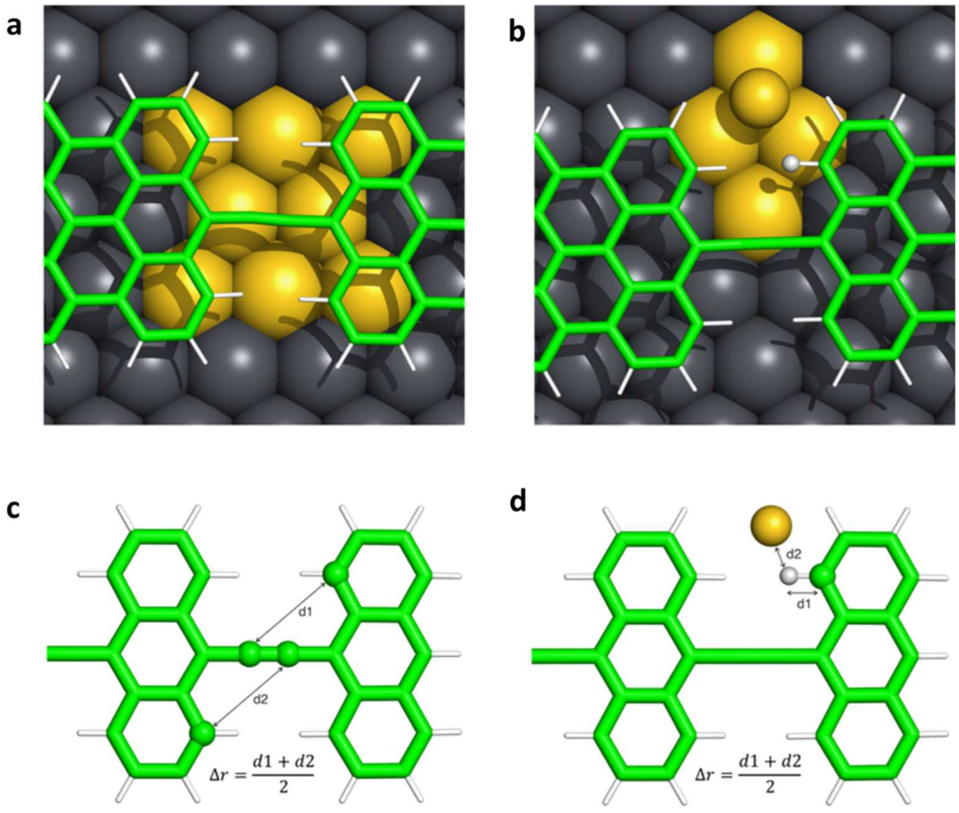

Supplementary Fig. 2 | Schematic view of QM/MM regions and reaction coordinates. Schematic view of partitioning of surface atoms belonging to QM region in the slab (yellow atoms) for the cyclization (**a**) and hydrogen abstraction (b), respectively. **c,** schematic view of the reaction coordinates of the cyclization mechanism. **d,** schematic view of the reaction coordinates of the hydrogen abstraction mechanism to a gold adatom.

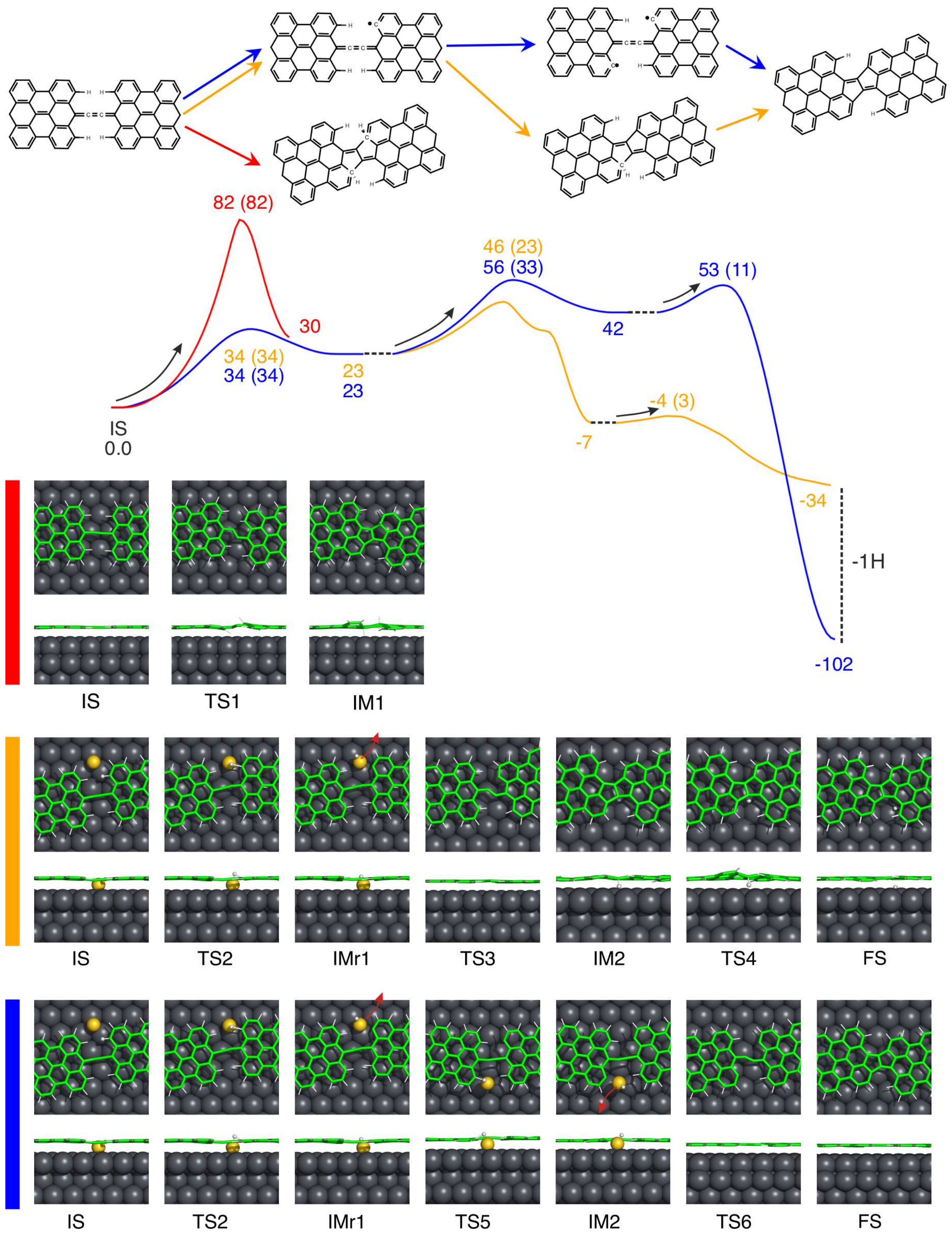

Supplementary Fig. 3 | Scheme of calculated reaction pathways of bisanthene polymer (2). Schematic view of distinct reaction pathways of transformation of cumulene-bridged bisanthene polymer to pentalene-bridged bisanthene including energy values (kcal/mol) of initial (IS), transition (TS), intermediates (IM) and final (FS) states. Values of the activation energies are presented in brackets. We considered 3 possible reaction pathways: (i) direct cyclization (red); (ii) hydrogen dissociation, cyclization and hydrogen dissociation (yellow); and (iii) hydrogen dissociation, hydrogen dissociation and cyclization (blue). Corresponding atomic arrangements of different states along the reaction pathways are shown below.

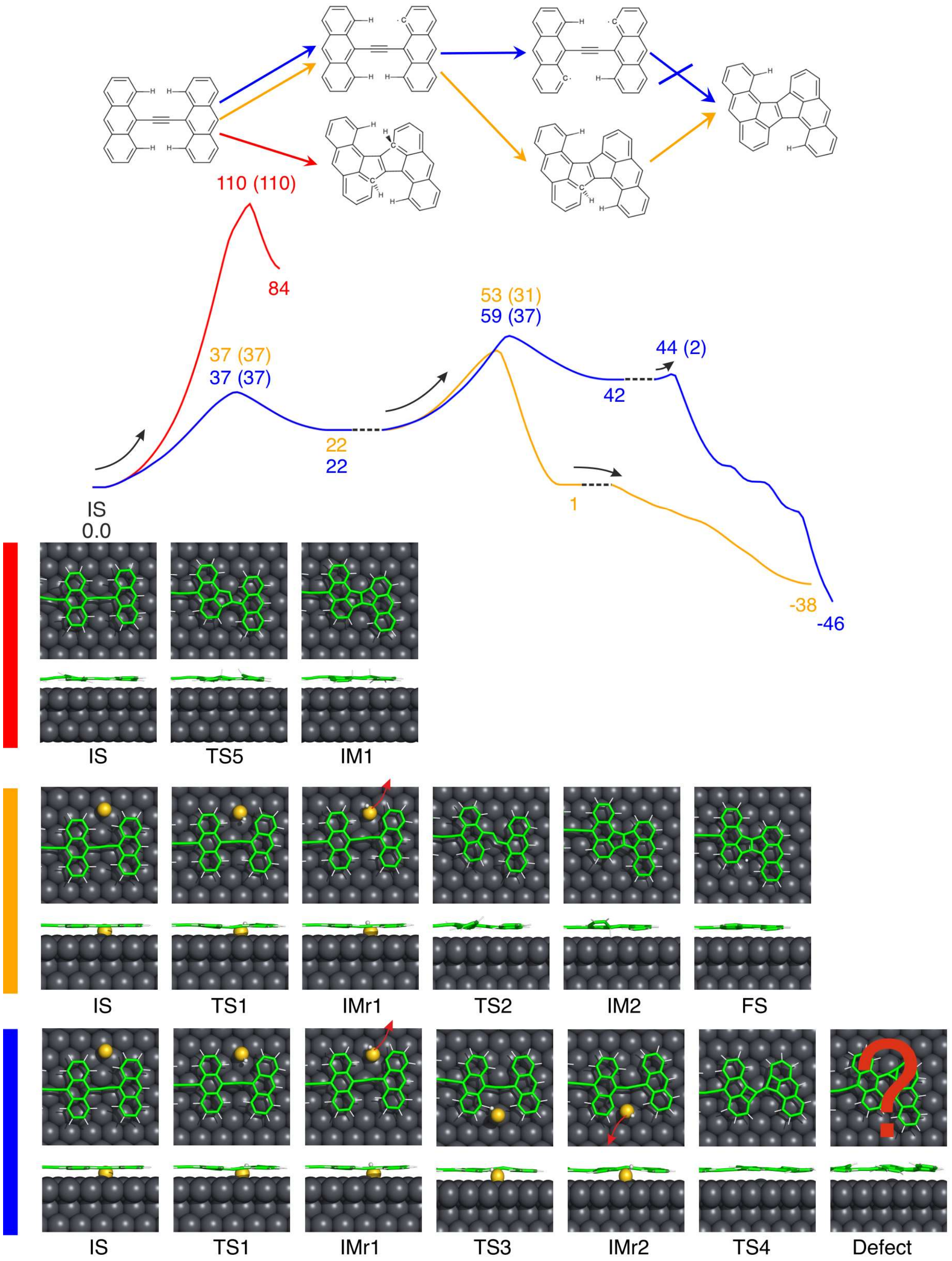

Supplementary Fig. 4 | Scheme of calculated reaction pathways of anthracene polymer (1). Schematic view of distinct reaction pathways of transformation of ethynylyne-bridged anthracene polymer to pentalene-bridged anthracene including energy values (kcal/mol) of initial (IS), transition (TS), intermediates (IM) and final (FS) states. Values of the activation energies are presented in brackets. We considered 3 possible reaction pathways: (i) direct cyclization (red); (ii) hydrogen dissociation, cyclization and hydrogen dissociation (yellow); and (iii) hydrogen dissociation, hydrogen dissociation and cyclization (blue). Corresponding atomic arrangements of different states along the reaction pathways are shown below. Note that in the case of the dissociation-dissociation-cyclization reaction pathway, other structures instead of pentalene-bridged anthracene polymer are preferred.

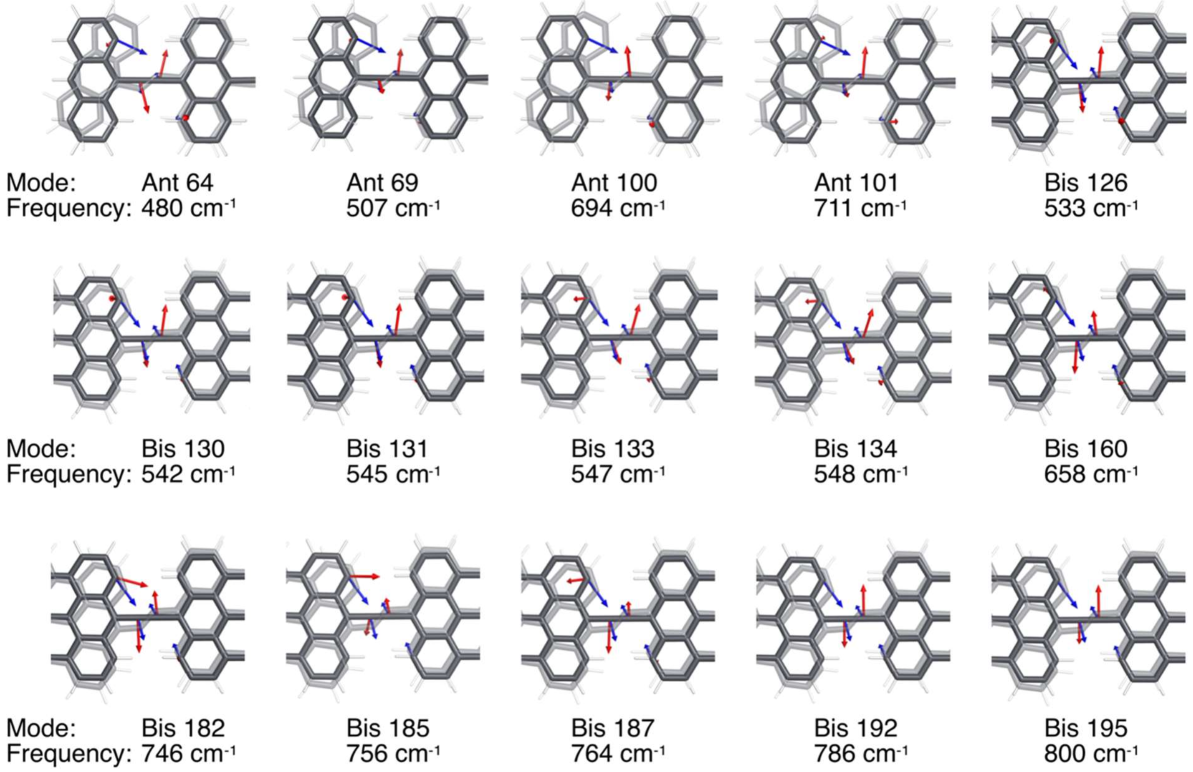

Supplementary Fig. 5 | List of available bending vibrational modes in anthracene and bisanthene polymers and their comparison with the reaction coordinates. We show selected vibrational modes with dominant contribution of the bending modes located on the bridging unit (corresponding vibrational eigenvectors are depicted by red arrows. Reaction coordinates (shown by blue arrows) are defined as the displacement from the initial (**IMr1**) to the transition state (**TS2**) for the cyclization reaction pathway from **IMr1** to **IM2**, see Supplementary Figs. 4 and 5. In the case of the anthracene polymer, none of the vibrational modes contains both the bending mode of the ethynylene bridge synchronized with characteristic in-phase motion of dehydrogenated carbon. The selected modes were selected from all vibrational modes obtained from the dynamical matrix DFT-Fireball calculations of free-standing IMr1 model consisting of four acene units.

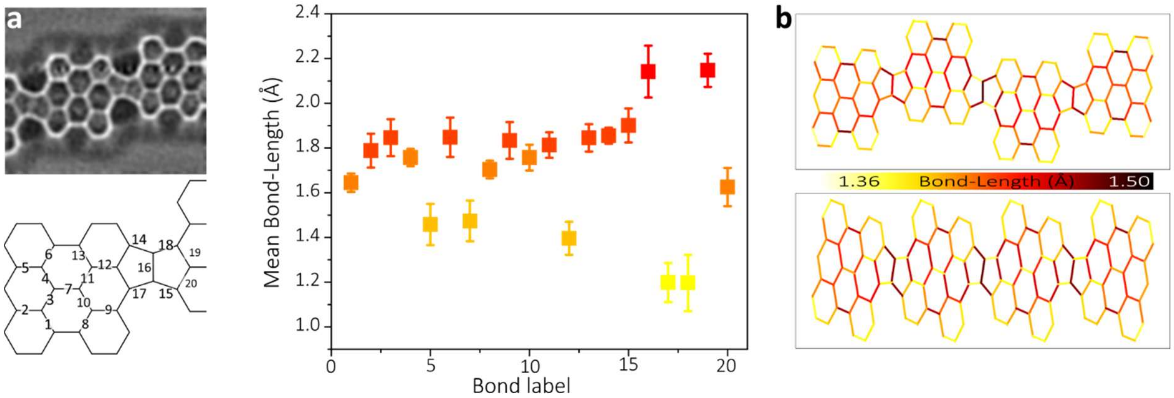

Supplementary Fig. 6 | Experimental and theoretical bond-length analyses. a, Typical nc-AFM high resolution image with applied Laplace-filtered to emphasise polymer structure used for obtaining experimentally C-C bond lengths (top-left panel) and C-C bond labelling (bottom left panel). Mean C-C bond-length plot obtained from a statistical analysis over several polymer structures. **b,** DFT calculated bond-length for *trans*- and *cis*- pentalene polymer displayed in a scale of colours.
